# Clinical and Radiographic Outcomes of Autologous Platelet-Rich Products in Regenerative Endodontics: A Systematic Review and Meta-Analysis

**DOI:** 10.3390/dj13060236

**Published:** 2025-05-26

**Authors:** Raewyn Huang, Wei Chen, Matthew Fang, Ove A. Peters, Sepanta Hosseinpour

**Affiliations:** School of Dentistry, The University of Queensland, Brisbane 4006, Australia; raewyn.huang@uq.net.au (R.H.); w.chen6@student.uq.edu.au (W.C.); m.fang@uq.net.au (M.F.); o.peters@uq.edu.au (O.A.P.)

**Keywords:** autologous platelet concentrates, platelet-rich plasma, platelet-rich fibrin, immature teeth, regenerative endodontic therapy

## Abstract

**Background:** Regenerative endodontic therapy (RET) allows for continued root development in necrotic immature permanent teeth. Autologous platelet concentrates (APC) such as platelet-rich plasma (PRP) and platelet-rich fibrin (PRF) are proposed alternatives to conventional blood clot scaffolds (BCS). **Objectives:** This systematic review compared the use of APC and BCS in RET of human necrotic immature permanent teeth. **Methods:** The electronic databases MEDLINE, CENTRAL and EMBASE were searched for randomized controlled trials (RCTs) which investigated the efficacy of APC and BCS in RET. We conducted this review following PRISMA 2020 guidelines. Quantitative radiographic outcomes of root length and root thickness were considered. The included RCTs were assessed for risk of bias using the Cochrane Risk-of-Bias tool. A meta-analysis was performed using RevMan 5.4. The protocol was registered on PROSPERO (CRD42023391536). **Results:** Of the 89 records screened, 10 RCTs were included in this review, in which 373 necrotic immature permanent teeth were treated. Both APC and BCS enable continued root development and would therefore be preferred over conventional root canal therapy with or without apexification. The meta-analysis revealed a statistically significant improvement in clinical success for PRP compared to BCS (Risk Ratio [RR] = 1.14, 95% Confidence Interval [CI]: 1.03–1.27, *p* = 0.02), whereas no significant difference was observed between PRP and PRF (RR = 1.04, 95% CI: 0.95–1.13, *p* = 0.37). **Conclusions:** PRP and PRF scaffolds enhance root length and thickness development in REPs compared to BCS. While PRP provides a modest but significant improvement in clinical success over BCS, PRF and PRP exhibit similar clinical outcomes. These findings support the use of platelet concentrates as viable alternatives to conventional blood clot scaffolds in regenerative endodontics.

## 1. Introduction

Immature necrotic permanent teeth are difficult to treat, because the open apex increases the risk of root canal irrigant extrusion and is not conducive to the placement and compaction of obturation materials. These teeth are conventionally treated by apexification to close the open apex, followed by root canal chemomechanical debridement and obturation. Apexification resolves infection with a high success rate, but the root ceases to mature, with potential complications such as inflammatory and replacement resorption. The immature root remains short in length with thin dentinal walls, which predisposes it to root fracture and potentially an inadequate crown-to-root ratio for future restorative treatment [1]. Regenerative endodontic therapy (RET) attempts to address these limitations by regenerating the pulp–dentine complex, which facilitates continued root development and restores the ability of the pulp to physiologically adapt to future insults [2].

RET relies on the combined presence of stem cells, growth factors, and scaffolds. The stem cells may either be externally introduced into the root canal, or may migrate from existing stem cell pools in the apical papilla or periodontal ligament [3,4]. The conventional scaffold is a blood clot, which is induced by instrumenting beyond the immature root apex. Platelets in the blood clot scaffold (BCS) release growth factors which regulate cell growth. Whilst BCS demonstrates evidence of continued root development [5], it is limited in its low and unpredictable concentration of growth factors. The erythrocytes in the BCS subsequently undergo necrosis and create a proinflammatory microenvironment [6], which may lead to unpredictable treatment outcomes [7]. An alternative approach is the use of autologous platelet concentrates (APC) as a scaffold. Compared to BCS, APC contains a higher concentration of platelets and growth factors, including but not limited to platelet-derived growth factor and vascular endothelial growth factor, which may increase the success rate and predictability of RET outcomes [6,8]. APC also contains a substantially lower concentration of erythrocytes than BCS.

The two formulations of APC which have been considered for RET are platelet-rich plasma (PRP) and platelet-rich fibrin (PRF). PRP is prepared from anticoagulated blood by double centrifugation and requires the addition of a platelet activator and an anticoagulant antagonist before use [9,10]. PRP is injected into the root canal and subsequently clots. PRF is prepared from non-anticoagulated blood by single centrifugation, without any additions of anticoagulants, platelet activators or anticoagulant antagonists. During centrifugation, platelets are activated and become trapped within a fibrin clot [9]. The fibrin clot is retrieved and placed into the root canal. PRF is simpler and cheaper to prepare than PRP, as no biochemical additions are necessary [9]. However, since platelet activation begins during centrifugation, PRF preparation is more technique-sensitive and the prepared product must be used immediately [9].

The primary advantage of APC over BCS is the elevated platelet concentration. These platelets contain multiple synergistically working growth factors in physiological concentrations, which facilitate chemotaxis, mitogenesis, and stem cell differentiation [6,10]. Compared to platelet concentrates prepared by blood banks sourced from allogeneic donors, APC is simple to harvest, abundant in supply, and can be prepared at the time of the surgery. Importantly, their autologous nature avoids the risk of immunogenic reactions and cross-infection to the patient [10].

Histological evidence from in vivo studies indicates that following APC treatment, cementum-like and bone-like tissues form on the internal wall of the root canal, and fibrous connective tissues form in the canal space [11]. Although true regeneration of pulpal and dentinal tissues is not achieved, the increased root thickness and root length would improve root structural strength.

The ideal method for evaluating the efficacy of APC would involve histological and mechanical testing of treated human teeth; however, such assessments are largely impractical in clinical studies. As an alternative, randomized controlled trials (RCTs) provide valuable evidence by radiographically measuring the extent of continued root development. While numerous RCTs have investigated this topic, there remains a lack of high-quality systematic reviews and meta-analyses directly comparing the efficacy of APC and BCS in RET for necrotic immature permanent teeth. This systematic review and meta-analysis aims to address this gap by assessing the clinical outcomes of APC application, focusing on root length and root thickness as key outcome parameters.

## 2. Methods

This review was conducted in accordance with the 2020 Preferred Reporting Items for Systematic Reviews and Meta-Analyses (PRISMA) guidelines [12] and has been registered in PROSPERO (CRD42023391536).

### 2.1. Search Strategy

The research question of this review was based on the PICOS framework: Population: human necrotic immature permanent teeth; Intervention: autologous platelet concentrates, including platelet-rich plasma and platelet-rich fibrin; Comparison: regenerative endodontic therapy using blood clot scaffolds; Outcomes: root length and root thickness; Study design: randomized controlled trials.

An electronic search was undertaken in three digital databases inclusive of MEDLINE, CENTRAL and EMBASE from January 2000 to January 2025. The search strategy followed the Peer Review of Electronic Search Strategies (PRESS) checklist [13]. The search terms included (“platelet-rich products” OR “autologous platelet concentrates” OR “platelet-rich plasma” OR “platelet-rich fibrin”) AND (“blood clot” OR “induced bleeding”) AND (endodontic* AND regenerat*) OR (pulp AND (revitali* OR revasculari* OR regenerat*)) OR “apical closure” OR (root AND (length* OR thick*))). The search was conducted on titles, abstracts and keywords only. No restrictions were placed on the date of publication, but the search was restricted to English publications. In addition to the electronic search, we also performed manual citation searching of the reference lists of included studies and relevant reviews to identify any additional eligible studies.

### 2.2. Study Selection

This review included RCTs published in English that investigated the treatment of immature necrotic permanent teeth in human subjects. Eligible studies compared an experimental group receiving APC with a control group receiving BCS. The primary outcome variables assessed were root length and root thickness, measured quantitatively as either absolute or proportional change.

Studies were excluded if they were in vivo or in vitro studies, case reports, case series, or non-randomized clinical trials. Research involving vital or mature teeth was also excluded. Additionally, studies that did not assess root length or root thickness, or those that measured outcomes qualitatively using descriptors such as “no change”, “fair”, “good”, or “excellent”, were not considered. Although qualitative measurements may reflect the efficacy of treatment, they are by nature subjective, with high intra- and inter-assessor variability, and hence are not conducive to comparison across studies.

The titles and abstracts of all studies retrieved from the search were downloaded onto EndNote 20.4.1. Duplicates were identified and removed. Two review authors independently assessed the titles and abstracts of the retrieved studies. The full texts of studies which appeared to meet inclusion criteria and those which had insufficient information in the title and abstract were retrieved. The full text reports were independently assessed by the two review authors. Any disagreements on the eligibility of included studies were resolved by discussion between the two review authors and consultation with the third review author when required.

### 2.3. Data Extraction

Data from the included studies were extracted independently by two review authors using a data extraction form. Any differences were resolved by discussion between the two authors.

We extracted the characteristics of the included studies, including the sample size, follow-up information and the sex and age of the participants. We extracted the methodology of the included studies, including the disinfection protocol, production of APCs, authenticity of APCs, the BCS procedure, and the follow-up protocol. We extracted the results on the clinical and radiographical outcome (root length and root thickness) from the included studies.

### 2.4. Assessment of the Risk of Bias

Two authors independently evaluated the risk of bias in the included studies using the revised Cochrane risk-of-bias tool for randomized trials [14]. Any discrepancies were resolved through discussion between the authors.

The risk of bias (RoB) assessment was graded as “high”, “some concerns” or “low” in each of the following five domains: the randomization process, deviations from the intended interventions, missing outcome data, measurement of the outcome and selection of the reported result. A study was judged at low RoB if all domains are at low RoB; at some concerns if at least one domain has some concerns; and at high RoB if at least one domain is at high RoB, or if multiple domains have some concerns in a manner that challenges the confidence in the results.

### 2.5. Results Analysis

The meta-analyses were conducted using Review Manager software (RevMan 5.4, The Cochrane Collaboration, Copenhagen, Denmark). Success rates were assessed using risk ratios (RRs), while mean changes in root length and root thickness were analyzed using mean differences (MDs), both with corresponding 95% confidence intervals (CIs). A random-effects model was applied, as it provided a more conservative estimate by accounting for variability across studies.

## 3. Results

### 3.1. Study Selection

**Figure 1:**
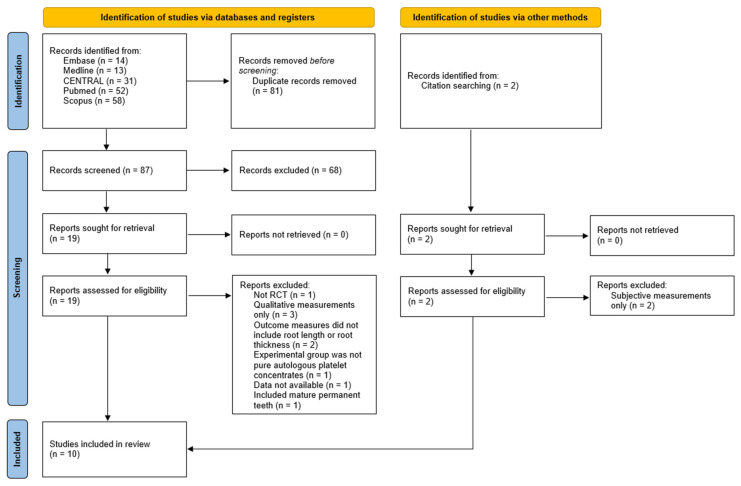
PRISMA flow chart summarizing study selection.

Figure 1 outlines our study selection process. A total of 168 studies were identified from our search strategy. Two additional studies were identified via citation searching. After the removal of duplicates and irrelevant studies, 19 studies were assessed for eligibility according to inclusion and exclusion criteria. Ten RCTs [15,16,17,18,19,20,21,22,23,24] were included in our review. Eight studies were excluded for the following reasons: root length and root thickness were measured subjectively [25,26,27,28]; root length and root thickness were not assessed [29], mature permanent teeth were tested [30], there was no APC only group [31]; and publication as a conference paper [32].

### 3.2. Characteristics of Included Studies

A total of 379 participants and 373 necrotic, immature permanent teeth were treated. Table 1 describes the patient demographics and characteristics of the included studies. The studies were conducted in Egypt, Turkey, Saudi Arabia, and India, with the majority focusing on maxillary incisors affected by dental trauma. Some studies also included mandibular incisors and premolars, with pulp necrosis attributed to either trauma or caries.

Blood was typically drawn intravenously from the antecubital vein, with or without anticoagulants such as dipotassium EDTA, calcium citrate, or acid citrate dextrose. Centrifugation procedures varied widely, with some studies employing single-step protocols (e.g., 3000 rpm for 10 min) [20,23,24] while others used multi-step sequences to optimize the scaffold composition [19,22,24]. Some studies fragmented PRF before placement [17,20,23], while others incorporated PRP into a sterile collagen sponge for enhanced apical positioning [16,20,22,24] (Table 2).

Sodium hypochlorite (NaOCl) was the primary irrigant, with concentrations ranging from 1.25% to 5.25%, followed by 20 mL of sterile saline and 10–20 mL of 17% EDTA in most studies. Antibiotic use varied across studies. Most studies used a triple antibiotic paste (metronidazole, ciprofloxacin, and minocycline) at a concentration of 0.1 mg/mL, while a few omitted antibiotics entirely. One study used a different combination, including clindamycin (Table 3).

#### Quality Assessment of Included Studies

**Figure 2:**
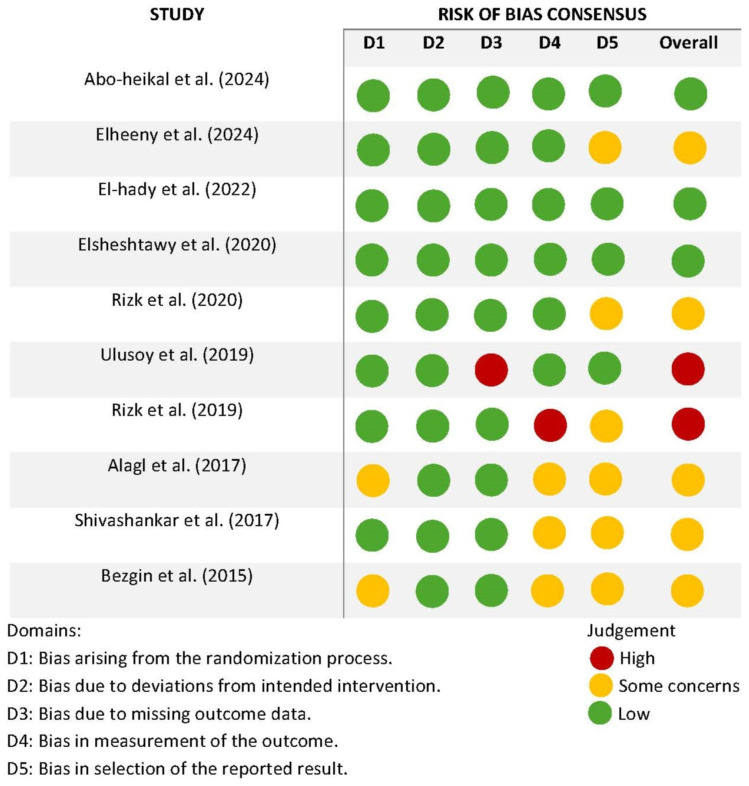
Risk of bias summary. Included studies: Abo-heikal et al. 2024 [19], Elheeny et al. 2024 [17], El-hady et al. (2022) [23], Elsheshtawy et al. (2020) [16], Rizk et al. 2020 [21], Ulusoy et al. 2019 [20], Rizk et al. 2019 [24], Alagl et al. 2017 [22] Shivashankar et al. 2017 [15], Bezgin et al. 2015 [18].

The risk of bias (RoB) assessment for the included studies is summarized in Figure 2, evaluating five domains. Most studies demonstrated a low risk of bias in domains D1 to D4, particularly those published from 2020 onward, indicating improved methodological rigor in recent years. However, concerns regarding selective reporting (D5) were observed in multiple studies, including Elheeny et al. [17], Alagl et al. [22], Shivashankar et al. [15], and Bezgin et al. [18]. Two studies, Rizk et al. [24] and Ulusoy et al. [20], had high-risk judgments in the measurement of outcomes (D4) and deviations from intended intervention (D2), affecting the overall reliability of their findings.

### 3.3. Clinical Outcomes

The clinical success rates of the selected studies are summarized in Table 4. The pre-operative periapical diagnoses varied among studies, with some reporting specific conditions such as symptomatic apical periodontitis, chronic apical periodontitis, and periapical abscesses [17,19], while others did not provide detailed diagnostic classifications. Despite these variations, all included studies reported high clinical success rates, with most cases achieving 90–100% success regardless of the scaffold material used. Complete apical closure was inconsistently reported [18,20,22] but showed variable outcomes, ranging from 50% to 100%. Pulp sensibility responses at 6 and 12 months were more variable, with some studies reporting no positive responses [15,16,21,24], while others showed progressive improvement over time [18,19,20,22]. PRF and PRP scaffolds generally demonstrated better pulp sensibility responses than blood clot scaffolds (BCS), particularly at 12 months.

### 3.4. Radiographic Outcomes

The root length and root thickness results of the selected studies are presented in Table 5 and Table 6, respectively.

Alagl et al. [22] reported a statistically significant advantage of PRPs over BCS in root length increases, and Rizk et al. [24] reported a statistically significant advantage of PRPs over BCS in both root length and thickness increases. The mean (SE) difference in absolute root length increases between PRP and BCS in these two studies ranged between 0.6 (0.2) mm and 0.8 (0.2) mm. The mean (SE) difference in percentage root length increases between PRP and BCS was 5% in Rizk et al. [24] and was not reported in Alagl et al. [22]. The mean (SE) difference in absolute root thickness increases between PRP and BCS in Rizk et al. [24] was 0.3 (0.3) mm and the mean (SE) difference in percentage root thickness increase was 14%.

Only Rizk et al. [21] reported a statistically significant advantage of PRF over BCS in root length increases. The mean (SE) difference in absolute root length increases between PRF and BCS in this study was 0.6 (0.2) mm. The mean (SE) difference in percentage root length increase was 4%. Notably, all studies using PRF showed no statistically significant difference in root thickness change compared to BCS.

Rizk et al. [21] reported a higher proportion of crown discoloration in the BCS group, but did not report the incidence of this.

**Figure 3:**
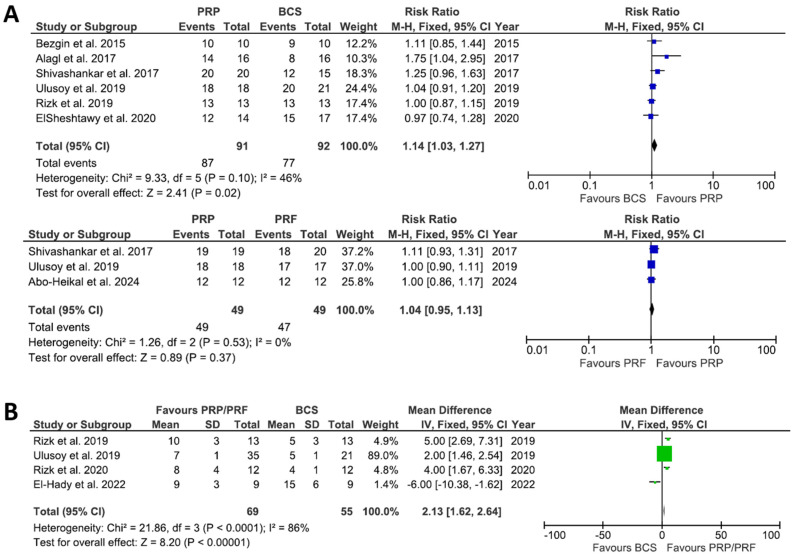

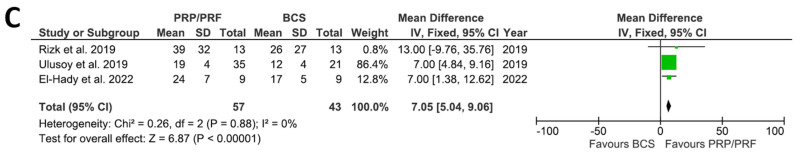
Forest plots from meta-analyses comparing autologous platelet concentrates (APC) with blood clot scaffolds (BCS) for (**A**) clinical success (primary outcome), (**B**) increase in root length, and (**C**) increase in root thickness. The forest plots show the effect size (risk ratio or mean difference) with 95% confidence intervals for each included study and the overall pooled effect. Abo-heikal et al. 2024 [19], Elheeny et al. 2024 [17], El-hady et al. (2022) [23], Elsheshtawy et al. (2020) [16], Rizk et al. 2020 [21], Ulusoy et al. 2019 [20], Rizk et al. 2019 [24], Alagl et al. 2017 [22] Shivashankar et al. 2017 [15], Bezgin et al. 2015 [18].

Figure 3 represents the results of the meta-analysis comparing PRP/PRF to BCS. Panel A shows the risk ratio (RR) for clinical success, with the first forest plot indicating a statistically significant advantage of PRP over BCS (RR = 1.14, 95% CI: 1.03–1.27, *p* = 0.02), while the comparison between PRP and PRF showed no significant difference (RR = 1.04, 95% CI: 0.95–1.13, *p* = 0.37). Panel B illustrates the mean difference in root length gain, showing an increase in root development for PRP/PRF compared to BCS (MD = 2.13, 95% CI: 1.62–2.64, *p* < 0.00001), despite substantial heterogeneity (I^2^ = 86%). Panel C presents the mean difference in root width gain, demonstrating an improvement in root dentin thickness for PRP/PRF compared to BCS (MD = 7.05, 95% CI: 5.04–9.06, *p* < 0.00001), with low heterogeneity (I^2^ = 0%).

## 4. Discussion

This systematic review analyzed ten RCTs comparing the efficacy of APC and BCS in increasing root length and root thickness following RET in necrotic immature permanent teeth. Unlike previous systematic reviews that included non-randomized studies, this review exclusively analyzed randomized controlled trials to minimize bias and ensure methodological rigor.

### 4.1. Comparison of PRP, PRF, and BCS

The meta-analysis demonstrated that PRP improved clinical success rates over BCS (RR = 1.14, 95% CI: 1.03–1.27, *p* = 0.02), whereas PRF showed no additional benefit over PRP. Additionally, PRP/PRF significantly increased root length (MD = 2.13 mm, 95% CI: 1.62–2.64, *p* < 0.00001) and root width (MD = 7.05 mm, 95% CI: 5.04–9.06, *p* < 0.00001) compared to BCS. The observed improvement in root development with PRP/PRF could be attributed to their ability to release bioactive growth factors that promote cell migration, proliferation, and differentiation. However, the clinical significance of these differences remains debatable. While Haralur et al. [35] demonstrated that increasing root thickness from 3.0 mm to 5.0 mm enhances fracture resistance by 70%, the mean root thickness gain in this review (approximately 7 mm) suggests a potential but not definitive advantage of PRP/PRF over BCS. The included studies reported a mean root length absolute difference of 0.4 mm to 0.8 mm and percentage difference of 0% to 5% between APC and BCS. If a fixed prosthesis is required and a crown root ratio of 1:2 is adhered to [36], this difference in root length would permit an additional 0.1 mm to 0.3 mm of prosthesis crown height. This difference may lead to an increased fracture resistance of approximately 10%. Therefore, the observed benefit in root length and thickness of APC over BCS may not be clinically significant.

### 4.2. Outcome Assessment and Methodological Considerations

The included studies varied in their assessment criteria. Some defined success primarily by radiographic evidence of healing and root development, while others incorporated pulp vitality testing (e.g., Ulusoy et al. [20]). Although periapical radiographs were the most common imaging modality, they are prone to geometric distortion. The lack of standardization in outcome assessment may have influenced the variability in reported success rates. Given the small differences in effect sizes, high precision is required to measure the difference between the experimental and control groups. Algal et al. [22], Abo-Heikal et al. [19], Elheeny et al. [17], and Elsheshtawy et al. [16] measured root length and root thickness using CT and CBCT, while all other studies [18,20,23,24] used intraoral periapical radiographs (PA) only. PA is prone to geometric distortion due to the variation in angulation of the radiographic film and the lack of a scale [37]. These intrinsic limitations of PA reduce the precision of the outcome measurements. This is reflected in the large standard errors of mean differences in most results.

The RoB varied amongst the included studies. Only two RCTs had low RoBs [20,24], while others had unclear or high RoB due to limitations in allocation concealment and blinding of outcome assessors. Given the nature of REPs, blinding is challenging beyond the initial procedure; however, adherence to pre-specified protocols and standardized assessment criteria could improve study reliability. Due to the nature of the treatment, participants and operators can only be blinded up to the appointment in which APC or BCS is administered. Since outcome measures of root length and root thickness are not subjective to the patient’s perception of the treatment, the patient placebo effect is likely minimal. Nonetheless, studies ought to adhere to the concealment of allocation sequence prior to APC or BCS administration and the blinding of outcome assessors. A pre-specified study protocol and analysis plan could only be found for the studies by El-Hady et al. [23] and Ulusoy et al. [20], and hence we cannot exclude the possibility that the methodology of the other studies were altered during intervention, rather than in accordance with a pre-specified plan.

### 4.3. Variability in REP Protocols

Disparities in irrigation protocols and platelet concentrate preparation methods may have contributed to outcome variability. Endodontic irrigants can release and activate growth factors within the dentine matrix which can enhance the migration, proliferation, and differentiation of stem cells from the apical papilla (SCAP) [37]. However, this must be balanced against the risk of cytotoxicity of the irrigants to the SCAP that enter the canal space during RET. 1.5% NaOCl irrigant solution has been recommended and subsequent irrigation with 17% EDTA has been shown to promote the survival of SCAP, most likely due to its neutralizing action against the cytotoxic effects of NaOCl [37,38]. Additionally, the sequential use of NaOCl and EDTA irrigation has been found to favor the release of transforming growth factor beta 1 [39]. Antibiotic selection may also impact regenerative outcomes. The most commonly used combination was a 1:1:1 mix of metronidazole, ciprofloxacin, and minocycline at 0.1 mg/mL, which is in line with current RET guidelines. However, one study substituted clindamycin, and several others did not use antibiotics. The absence or substitution of antibiotics may reduce bacterial elimination or alter stem cell viability, thereby impacting clinical success and tissue regeneration. Future studies should aim to standardize antibiotic protocols or evaluate their necessity in APC-based RET.

Given the inconsistency in APC scaffold production techniques reported in RCTs included in this review [15,16,17,18,19] it would be beneficial to investigate the authenticity of the APC being produced. Boswell et al. [39] showed that failure to concentrate platelets is not uncommon, and they suggested that a complete blood count should be conducted on both the whole blood and the APC to ensure that the APC has properly concentrated platelets. No existing RCT conducted such a complete blood count, so it is possible that the results obtained from these studies may not reflect the true regenerative potential of PRP and PRF scaffolds.

This review is limited by the small number of eligible studies and the variability in study protocols. The heterogeneity in outcome assessment methods and APC production techniques may have influenced the findings. Additionally, the lack of long-term follow-up data restricts conclusions regarding the durability of treatment success. A broader meta-analysis including well-designed cohort studies may provide further clarity, though at the expense of increased bias.

## 5. Conclusions

The use of APC demonstrates an advantage over BCS in terms of clinical success and root development, while PRF does not offer additional benefits over PRP in terms of root development. However, the limited number of high-quality RCTs with standardized methodologies and outcome measures hinders a definitive conclusion on APC efficacy. The standardization of REPs, including APC preparation and outcome assessment, is essential for future studies to establish best practices in regenerative endodontics.

## Figures and Tables

**Table 1:** Participant characteristics of included studies.

Author (Year)	Study Design	Country	Teeth Type	Etiology of Pulp Necrosis	Scaffold	Number Participants (Number of Participants Lost to Follow-Up)	Number of Teeth (Number of Teeth Lost to Follow-Up)	Sex	Age Range (Years)	Follow-Up Protocol (Months)
Abo-Heikal et al. (2024) [19]	RCT, parallel	Egypt	Maxillary incisors	Dental trauma	PRF	12(0)	12(0)	9M, 3F	12–21	6, 12
					PRP	12(0)	12(0)	9M, 3F		
Elheeny et al. (2024) [17]	RCT, parallel	Egypt	Maxillary incisors	Dental trauma	PRF	27(1)	33(2)	16M, 11F	8–10	6, 12
					CGF	28(1)	33(2)	14M, 14F		
El-Hady et al. (2022) [23]	RCT, parallel	Egypt	Maxillary incisors	Dental trauma	PRF	NR(1) ^†^	10(1)	NR	8–12	3, 6, 12
					BCS	NR(1) ^†^	10(1)	NR		
ElSheshtawy et al. (2020) [16]	RCT, parallel	Egypt	Maxillary and mandibular incisors	Dental trauma/anomalies	PRP	13(0)	14(0)	5M, 8F	8–12	3, 6, 9, 12
					BCS	13(0)	17(0)	10M, 3F		
Rizk et al. (2020) [21]	RCT, split mouth	Egypt	Maxillary central incisors	Dental trauma	PRF	13(1)	13(1)	6M, 6F ^‡^	9.1 ± 1.2 ^§^	3, 6, 9, 12
					BCS	13(1)	13(1)	6M, 6F ^‡^		
Ulusoy et al. (2019) [20]	RCT, parallel	Turkey	Maxillary incisors	Dental trauma	PRP	88(15)	22(4)	8M, 10F	7–11	Variable, ranged from 10 to 49 months
					PRF		22(5)	10M, 7F		
					BCS		22(1)	13M, 8F		
Rizk et al. (2019) [24]	RCT, split mouth	Egypt	Maxillary central incisors	Dental trauma	PRP	13(0)	13(0)	7M, 6F	9.1 ± 1.0 ^§^	3, 6, 9, 12
					BCS	13(0)	13(0)	7M, 6F		
Alagl et al. (2017) [22]	RCT, split mouth	Saudi Arabia	Maxillary incisors and maxillary/mandibular premolars	Dental trauma /caries	PRP	16(1)	16(1)	10M, 6F	8–11	3, 6, 9, 12
					BCS	16(1)	16(1)	10M, 6F		
Shivashankar et al. (2017) [15]	RCT, parallel	India	Maxillary and mandibular incisors	Dental trauma /caries	PRF	20(0)	20(0)	32M, 28F	6–28	3, 6, 9, 18
					PRP	20(1)	20(1)			
					BCS	20(5)	20(5)			
Bezgin et al. (2015) [18]	RCT, parallel	Turkey	Maxillary incisors and maxillary/mandibular premolars	Dental trauma /caries	PRP	11(1)	11(1)	7M, 3F	7–12	3, 6, 9, 18
					BCS	11(1)	11(1)	4M, 6F		

Abbreviation: BCS: blood clot scaffold; CGF: concentrated growth factor, F: female, M: male, NR: not reported; PRF: platelet-rich fibrin; PRP: platelet-rich plasma; RCT: randomized controlled trial. ^†^ A total of 15 participants completed the study, but the number of participants in each group was not stated. ^‡^ One participant was lost to follow-up, sex was not stated. ^§^ Age of participants was reported as mean ± standard deviation.

**Table 2:** APC scaffold production in included studies.

Author (Year)	APC Scaffold	Blood Withdrawal	Anticoagulant	Centrifugation Procedure	Activation Agent/Anticoagulant Antagonist	Application of Scaffold
Abo-Heikal et al. (2024) [19]	PRP	10 mL of intravenous blood from the antecubital vein	Di-Potassium EDTA	Two rounds (2400 rpm for 10 min and 3600 rpm for 15 min)	10% calcium chloride	Injected into canal up to CEJ.
	Injectable–PRF		N/A	700 rpm for 3 min	10% calcium chloride	Injected into canal up to CEJ.
Elheeny et al. (2024) [17]	PRF	10 mL intravenous blood from antecubital vein	N/A	3000 rpm for 10 min	N/A	Fractions were inserted via hand small plugger up to 3 mm below the CEJ
	CGF		N/A	Acceleration for 30 s, 2 min at 2700 rpm, 4 min at 2400 rpm, 4 min at 2700 rpm, 3 min at 3000 rpm,	N/A	
El-Hady et al. (2022) [23]	PRF	Standard venipuncture withdrawn from medial cubital vein	N/A	1400 rpm for 14 min	N/A	PRF scaffold fragmented and placed in canal with a finger plugger.
ElSheshtawy et al. (2020) [16]	PRP	Intravenous blood	Calcium citrate	NR	NR	Injected into canal up to CEJ.
Rizk et al. (2020) [21]	PRF	5 mL intravenous blood from antecubital vein	N/A	2400 rpm for 12 min	N/A	Fractions were inserted via hand small plugger up to the CEJ
Ulusoy et al. (2019) [20]	PRP	20 mL intravenous blood	15 mL citrate	1250 rpm for 15 min	NR	PRP placed into canal to a level 3 mm below CEJ.
	PRF	10 mL intravenous blood from antecubital vein	N/A	3000 rpm for 10 min	N/A	Fractions were inserted via hand small plugger up to 3 mm below the CEJ.
Rizk et al. (2019) [24]	PRP	4.5 mL of intravenous blood from the antecubital vein	0.5 mL acid citrate dextrose	2400 rpm for 10 min, followed by 3600 rpm for 15 min	10% calcium chloride	PRP soaked on sterile collagen sponge and pushed beyond apical region and flush with the CEJ.
Alagl et al. (2017) [22]	PRP	Intravenous blood withdrawn ^†^	Not stated ^†^	Two rounds of centrifugation ^†^	100 U/mL bovine thrombin/10% calcium chloride	Injected into canal up to CEJ.
Shivashankar et al. (2017) [15]	PRP	10 mL of intravenous blood	NR	NR	NR	NR
	PRF		N/A	NR	NR	NR
Bezgin et al. (2015) [18]	PRP	Intravenous blood	NR	NR	10% calcium chloride	Injected into canal up to CEJ.

Abbreviations: APC: autologous platelet concentrate, CGF: concentrated growth factor, CEJ: cemento-enamel junction; min: minute, N/A: not applicable; NR: not reported; PRP: platelet-rich plasma; PRF: platelet-rich fibrin, rpm: revolutions per minute, sec: second. ^†^ Followed Dohan et al. [9]’s protocol for PRP production, but did not explicitly describe the blood withdrawal, anticoagulant use, and centrifugation steps. These steps were recorded from Dohan et al. [9]. However, Dohan et al. [9] did not describe these steps in detail; namely, the volume of blood drawn, the type of anticoagulant used, and the centrifugation settings were not described.

**Table 3:** Disinfection protocol and blood clot scaffold production in included studies.

Author (Year)	Canal Disinfection		Blood Clot Scaffold Production
	Irrigant	Antibiotic	
Abo-Heikal et al. (2024) [19]	20 mL 1.5% NaOCl, 20 mL sterile saline, 10 mL 17% EDTA	No antibiotics were used.	No blood clot.
Elheeny et al. (2024) [17]	20 mL 1.5% NaOCl for 5 min, 20 mL sterile saline for 5 min, 10 mL 17% EDTA	No antibiotics were used.	No blood clot.
El-Hady et al. (2022) [23]	20 mL 1.5% NaOCl for 5 min, 20 mL saline for 5 min	No antibiotics were used.	Size 25# K-file rotated 2 mm past apical foramen to induce bleeding up to CEJ.
ElSheshtawy et al. (2020) [16]	20 mL 2.5% NaOCl, 20 mL sterile saline, 10 mL 17% EDTA	0.1 mg/mL mixture of 1:1:1: metronidazole, ciprofloxacin, minocycline	Size 20# K-file rotated 2 mm past apical foramen.
Rizk et al. (2020) [21]	2% NaOCl, 17% EDTA	0.1 mg/mL mixture of 1:1:1: metronidazole, ciprofloxacin, minocycline	Unknown sized file used past periapical area to induce bleeding slightly below CEJ.
Ulusoy et al. (2019) [20]	20 mL 1.25% NaOCl	60 mg/mL mixture of 1:1:1 clindamycin: ciprofloxacin, metronidazole	Size 15# K-file passed 1–2 mm beyond apex to induce bleeding to at least 3 mm below CEJ. The tooth was allowed to clot for 10 min.
Rizk et al. (2019) [24]	20 mL 2% NaOCl for 5 min, 20 mL 17% EDTA for 5 min	0.1 mg/mL mixture of 1:1:1: metronidazole, ciprofloxacin, minocycline	Unknown sized file used to irritate periapical tissues to induce bleeding up to a point 2 mm below CEJ. The blood was allowed to clot for 10–15 min.
Alagl et al. (2017) [22]	20 mL 2.5% NaOCl, 20 mL sterile saline, 10 mL 0.12% chlorhexidine	0.1 mg/mL mixture of 1:1:1: metronidazole, ciprofloxacin, minocycline	Size 20# K-file rotated 2 mm past apical foramen to induce bleeding up to CEJ. Blood was allowed to clot.
Shivashankar et al. (2017) [15]	5.25% NaOCl	0.1 mg/mL mixture of 1:1:1: metronidazole, ciprofloxacin, minocycline	Size 20# K-file rotated 2 mm past apical foramen.
Bezgin et al. (2015) [18]	20 mL 2.5% NaOCl, 20 mL sterile saline, 10 mL 5% EDTA, 10 mL 0.12% chlorhexidine	0.1 mg/mL mixture of 1:1:1: metronidazole, ciprofloxacin, minocycline	Size 20# K-file rotated past apical foramen.

Abbreviations: CEJ: cemento-enamel junction; EDTA: ethylene di-amine tetra-acetic acid; min: minute, NaOCL: sodium hypochlorite.

**Table 4:** Pre-operative pulp and periapical diagnosis and reported clinical outcomes of the included studies.

Study	Pre-Operative Periapical Diagnosis *	Clinical Success Rate (%) During Follow-Up	Complete Apical Closure (%)	Positive % Pulp Sensibility Response at 6 Months	Positive % Pulp Sensibility Response at 12 Months
Abo-Heikal et al. (2024) [19]	Healthy: 4.2%, chronic apical periodontitis: 29.2%, chronic periapical abscess: 20.8%, acute exacerbation of a chronic periapical lesion: 45.8%	PRF: 100 PRP: 100	NR	PRF: 27.3 PRP: 18.2	PRF: 36.4 PRP: 27.3
Elheeny et al. (2024) [17]	Symptomatic apical periodontitis: 47.5%, chronic apical abscess: 50.5%	PRF: 93.9 CGF: 93.9	PRF: 96.9 CGF: 100	NR	PRF: 48.5 CGF: 57.6
El-Hady et al. (2022) [23]	NR	PRF: 100 BCS: 100	NR	NR	PRF: 33.3 BCS: 22.2
ElSheshtawy et al. (2020) [16]	NR	PRP: 85.7 BCS: 88	NR	PRP: 0 BCS: 0	PRP: 0 BCS: 0
Rizk et al. (2020) [21]	NR	PRF: 100 BCS: 100	NR	PRF: 0 BCS: 0	PRF: 0 BCS: 0
Ulusoy et al. (2019) [20]	NR	PRP: 100 PRF: 100 BCS: 100	PRP: 66.7 PRF: 70.6 BCS: 76.2	PRP: 61.1 PRF: 72.2 BCS: 42.8	PRP: 72.2 PRF: 88.8 BCS: 68.1
Rizk et al. (2019) [24]	NR	PRF: 100 BCS: 100	NR	PRF: 0 BCS: 0	PRF: 0 BCS: 0
Alagl et al. (2017) [22]	NR	PRP: 100 BCS: 100	PRP: 93 BCS: 50	NR	PRP: 86.6 BCS: 40
Shivashankar et al. (2017) [15]	NR	PRP: 100 PRF: 90 BCS: 100	NR	NR	PRP: 15.8 PRF: 15 BCS: 13.3
Bezgin et al. (2015) [18]	NR	PRP: 100 BCS: 90	PRP: 70 BCS: 60	NR	PRP: 50 BCS: 20

* Based on the reported diagnostic terminology in the included studies. Abbreviation: NR: not reported.

**Table 5:** Reported radiographic outcomes: root length results of included studies.

Author (Year)	APC Scaffold	Radiographic Imaging Modality	Summary Statistic	Results at 12 Months
Abo-Heikal et al. (2024) [19]	PRF, PRP	CBCT scan, intraoral periapical radiographs	Mean % increase in average root length	PRF: 3.61 ± 1.73, PRP: 3.17 ± 1.66, *p* = 0.54
			Mean % decrease in average apical canal diameter	PRF: 18.52 ± 4.25, PRP: 13.62 ± 3.70, *p* = 0.008
Elheeny et al. (2024) [17]	PRF, CGF	CBCT scan, intraoral periapical radiographs	Mean (SD) absolute increase in root length (mm)	PRF: 1.16 (0.83) CGF: 2.42 (0.98), *p* < 0.001
El-Hady et al. (2022) [23]	PRF	Intraoral periapical radiographs	Mean (SD) absolute increase in root length (mm)	PRF: 1.5 (1.8 ^†^), BCS: 1.9 (1.6 ^†^) Mean (SE) difference = 0.4 (0.8), *p* = 0.69 ^‡^
			Mean (SD) percentage increase in root length (%)	PRF: 9 (3), BCS: 15 (6) Mean (SE) difference = 5 (2), *p* = 0.10
ElSheshtawy et al. (2020) [16]	PRP	CBCT scan, intraoral periapical radiographs	Mean change in root length	No difference between groups
Rizk et al. (2020) [21]	PRF	Intraoral periapical radiographs	Mean (SD) absolute increase in root length (mm)	BCS: 0.6 (0.2), PRF: 1.2 (0.5) Mean difference (SE) = 0.6 (0.2), *p* = 0.005
			Mean (SD) percentage increase in root length (%)	BCS: 4 (1), PRF: 8 (4) Mean difference (SE) = 4 (1), *p* = 0.005
Ulusoy et al. (2019) [20]	PRF, PRP	Intraoral periapical radiographs	Mean (SD) percentage increase in root length (%) ^§^	PRP: 5 (1), PRF: 7 (1), BCS: 5 (1) Mean difference (SE) for PRP vs. BCS = 0 (0.4), *p* > 0.05 Mean difference (SE) for PRF vs. BCS = 2 (0.5), *p* > 0.05
Rizk et al. (2019) [24]	PRP	Intraoral periapical radiographs	Mean (SD) absolute increase in root length (mm)	BCS: 0.7 (0.4), PRP: 1.5 (0.4) Mean difference (SE): 0.8 (0.2), *p* < 0.001
			Mean (SD) percentage increase in root length (%)	BCS: 5 (3), PRP: 10 (3) Mean difference (SE): 5 (1), *p* < 0.001
Alagl et al. (2017) [22]	PRP	CBCT scan	Mean (SD) absolute increase in root length (mm)	BCS: 0.5 (0.4), PRP: 1.1 (0.6) Mean difference (SE) = 0.6 (0.2), *p* = 0.004
Shivashankar et al. (2017) [15]	PRF, PRP	Intraoral periapical radiographs	Mean (SD) percentage increase in root length (%)	PRF: Not reported, PRP: not reported
Bezgin et al. (2015) [18]	PRP	Intraoral periapical radiographs	Increase in root area	PRP: 9.86%, BCS: 12.6%, *p* > 0.05

APC: autologous platelet concentrate; BCS blood clot scaffold; CBCT: cone-beam computed tomography; CT: computed-tomography; PRF: platelet-rich fibrin; PRP: platelet-rich plasma; SD: standard deviation; SE: standard error. ^†^ El-Hady et al. [23] did not report root length as the change from baseline, and instead only reported root length at each time point. The SD for change from baseline is calculated according to $SD_{c}=\sqrt{SD_{b}^{2}+SD_{f}^{2}-(2r\times SD_{b}\times SD_{f})}$, where SDc is the SD of change from baseline, SD_b_ is the SD at baseline, and SD_f_ is the SD post-intervention [33] (pp. 164–167). There was insufficient information from related studies to calculate the correlation coefficient, r, so r was estimated to be 0.7. ^‡^ El-Hady et al. [23] did not report the *p* value of the comparison of the means of APC and BCS groups. The *p* value was calculated according to Welch’s t-test, $t=\frac{{\bar{X}}_{1}-{\bar{X}}_{2}}{s_{\bar{∆}}}$, where $s_{\bar{∆}}=\sqrt{\frac{s_{1}^{2}}{n_{1}}+\frac{s_{2}^{2}}{n_{2}}}$, using a Student’s t-distribution with degrees of freedom $df=\frac{{\left(\frac{s_{1}^{2}}{n_{1}}+\frac{s_{2}^{2}}{n_{2}}\right)}^{2}}{\frac{{\left(s_{1}^{2}/n_{1}\right)}^{2}}{n_{1}-1}+\frac{{\left(s_{2}^{2}/n_{2}\right)}^{2}}{n_{2}-1}}$ [34]. ^§^ Ulusoy et al. [20] reported two analyses: with respect to follow-up time and irrespective of follow-up time. Since follow-up times varied between patients, results irrespective of follow-up are difficult to interpret and unlikely to be useful. Therefore, only results with respect to follow-up time are recorded.

**Table 6:** Reported radiographic outcomes: root thickness results.

Study	APC Scaffold	Radiographic Imaging Modality	Summary Statistic	Results at 12 Months
Abo-Heikal et al. (2024) [19]	PRF, PRP	CBCT scan, intraoral periapical radiographs	Mean % increase in average root thickness	PRF: 3.61 ± 1.58, PRP: 3.09 ± 1.44, *p* = 0.375
Elheeny et al. (2024) [17]	PRF, CGF	CBCT scan, intraoral periapical radiographs	Root dentin thickness (mm)	PRF: 5.71, CGF: 5.99
El-Hady et al. (2022) [23]	PRF PRP	Intraoral periapical radiographs	Mean (SD) absolute increase in root thickness at 1/3 of root thickness (mm)	PRF: 0.7 (0.6 ^†^), BCS: 0.7 (0.5 ^†^) Mean difference (SE) = 0 (0.3), *p* = 0.50 ^‡^
			Mean (SD) percentage increase in root thickness at 1/3 of root thickness (%)	PRF: 24 (7), BCS: 17 (5) Mean difference (SE) = 7 (3), *p* = 0.08
			Mean (SD) absolute increase in root thickness at 2/3 of root thickness (mm)	PRF: 0.7 (0.4 ^†^), BCS: 0.6 (0.5 ^†^) Mean difference (SE) = 0.1 (0.2), *p* = 0.32 ^‡^
			Mean (SD) percentage increase in root thickness at 2/3 of root thickness (%)	PRF: 28 (12), BCS: 18 (6) Mean difference (SE) = 10 (5), *p* = 0.14
ElSheshtawy et al. (2020) [16]	PRF	Intraoral periapical radiographs	Mean change in root dentinal thickness	No difference between groups
Rizk et al. (2020) [21]	PRF	Intraoral periapical radiographs	Mean (SD) absolute increase in root thickness (mm)	PRF: 0.9 (0.4), BCS: 0.7 (0.5) Mean difference (SE) = 0.2 (0.2), *p*= 0.117
Ulusoy et al. (2019) [20]	PRF, PRP	Intraoral periapical radiographs	Mean (SD) percentage increase in root thickness (%) ^§^	PRP: 19 (4), PRF: 11 (4), BCS: 12 (4) Mean difference (SE) for PRP vs. BCS = 7 (1), *p* > 0.05 Mean difference (SE) for PRF vs. BCS = −1 (1), *p* > 0.05
Rizk et al. (2019) [24]	PRP	Intraoral periapical radiographs	Mean (SD) absolute increase in root thickness (mm)	PRP: 1.0 (0.8), BCS: 0.7 (0.7) Mean difference (SE) = 0.3 (0.3), *p* = 0.002
			Mean (SD) percentage increase in root thickness (%)	PRP: 39 (32), BCS: 26 (27) Mean difference (SE) = 14 (12), *p* = 0.002
Bezgin et al. (2015) [18]	PRP	Intraoral periapical radiographs	Increase in root area	PRP: 9.86%, BCS: 12.6%, *p* > 0.05

BCS: blood clot scaffold; PRF: platelet-rich fibrin; PRP: platelet-rich plasma; SD: standard deviation; SE: standard error. ^†^ El-Hady et al. [23] did not report root thickness as the change from baseline, and instead only reported root thickness at each time point. The SD for change from baseline is calculated the same as in Table 5. ^‡^ El-Hady et al. [23] did not report the *p* value of the comparison of the means of APC and BCS groups. The *p* value was calculated the same as in Table 5. ^§^ Ulusoy et al. [20] reported two analyses: with respect to follow-up time and irrespective of follow-up time. Since follow-up times varied between patients, results irrespective of follow-up are difficult to interpret and unlikely to be useful. Therefore, only results with respect to follow-up time are recorded.
